# Are We Comparing Apples with Oranges? Assessing Improvement Across Symptoms, Functioning, and Goal Progress for Adolescent Anxiety and Depression

**DOI:** 10.1007/s10578-021-01149-y

**Published:** 2021-04-07

**Authors:** Karolin Rose Krause, Julian Edbrooke-Childs, Rosie Singleton, Miranda Wolpert

**Affiliations:** 1 Research Department for Clinical, Educational and Health Psychology, University College London, Gower Street, Bloomsbury, London, WC1E 6BT UK; 2 Evidence-Based Practice Unit, Anna Freud National Centre for Children and Families, 4–8 Rodney Street, London, N1 9JH UK; 3 Wellcome Trust, 215 Euston Rd, Bloomsbury, London, NW1 2BE UK

**Keywords:** Adolescents, Depression, Anxiety, Outcome, Functioning, Personalized measures

## Abstract

Strategies for comparing routinely collected outcome data across services or systems include focusing on a common indicator (e.g., symptom change) or aggregating results from different measures or outcomes into a comparable core metric. The implications of either approach for judging treatment success are not fully understood. This study drew on naturalistic outcome data from 1641 adolescents with moderate or severe anxiety and/or depression symptoms who received routine specialist care across 60 mental health services in England. The study compared rates of meaningful improvement between the domains of internalizing symptoms, functioning, and progress towards self-defined goals. Consistent cross-domain improvement was observed in only 15.6% of cases. Close to one in four (24.0%) young people with reliably improved symptoms reported no reliable improvement in functioning. Inversely, one in three (34.8%) young people reported meaningful goal progress but no reliable symptom improvement. Monitoring systems that focus exclusively on symptom change risk over- or under-estimating actual impact, while aggregating different outcomes into a single metric can mask informative differences in the number and type of outcomes showing improvement. A move towards harmonized outcome measurement approaches across multiple domains is needed to ensure fair and meaningful comparisons.

**Supplementary Information:**

The online version contains supplementary material available at 10.1007/s10578-021-01149-y.

## Introduction

Anxiety and depression are among the most common mental health conditions in young people worldwide [1–3]. In the absence of effective treatment, early-onset depression and anxiety can have significant adverse effects on mental health and socio-economic outcomes across the life course [4–7]. Treatments that have demonstrated efficacy in clinical trials [8–14] often do not yield the expected results in clinical practice [15, 16]. Routinely collected outcome data has an important role in enhancing the effectiveness of routine care for adolescent anxiety and depression, by informing adjustments to individual treatment plans, service planning, target setting and comparisons of effectiveness across settings and care models [17–20].

Services wishing to embed routine outcome monitoring face the challenge of having to identify the most important outcomes to measure, as well as the most suitable measurement instruments to track these outcomes. Notably, such instruments should be feasible, acceptable, valid, and reliable [21]. In addition, consultations with youth, families, and clinicians indicate that outcome measurement should be tailored to individual cases, and assess change holistically [22–24]. Services further face system-level challenges related to synthesizing and benchmarking data obtained from different measurement approaches. While standards for the routine measurement of outcomes for adolescent depression are beginning to emerge [21], they are not yet widely implemented.

One possible avenue for managing this challenge is to build reporting systems around a single outcome metric. For example, the US National Committee for Quality Assurance includes an indicator of symptomatic recovery derived from the Patient Health Questionnaire 9 (PHQ-9) [25] in its Healthcare Effectiveness Data and Information Set (HEDIS) to measure care quality for depression. Such approaches mirror common practice in clinical trials, where the designated primary outcome measure typically tracks symptom change [26]. While a single indicator provides the clarity that commissioners and policymakers require for decision-making and reporting purposes [27], there are questions about the extent to which a symptom score change by itself represents meaningful improvement in young people’s lives, including in their daily functioning [28–32].

A second avenue is to measure outcomes more holistically, across multiple domains (and possibly by using multiple measures within the same outcome domain), and to aggregate results into a standardized composite metric to simplify reporting and benchmarking (e.g., [33]). For example, young people could be considered “reliably improved” if they demonstrated reliable improvement on at least one of the individual measures or outcome domains considered, and if there was no reliable decline on any other [34]. Two outcome domains for consideration alongside symptoms are functioning and progress towards self-defined goals. Measures of functioning provide insight into how symptoms impact on daily life, and can anchor and contextualize symptom scores [32]. Progress towards self-defined goals is measured via a personalized scale where item content is determined by individual service users, enabling a person-centred progress assessment [35, 36].

The implications of either approach for determining treatment effectiveness for individual cases, and at a service level, are not well understood. There is limited evidence about the extent to which measures that purport to capture the same construct converge in their ratings for individual cases, or the extent to which improvement in one outcome domain translates into improvement in another domain [37, 38]. Existing research suggests that symptom change often exceeds change in functioning [39–41], but tends to be inferior to subjective perceptions of change or progress towards self-defined goals, as measured by personalized instruments [42, 43].

Existing studies have tended to use cross-diagnostic samples, and the extent to which their findings apply to adolescent anxiety and depression is unclear. There is some evidence that externalizing disorders are associated with higher functional impairment than internalizing disorders [44, 45], and the association between changes in symptoms and functioning may vary across clinical presentations. In addition, only two studies have examined the convergence of change ratings across different outcome domains at an individual level, and both were limited by small samples of around 120 cases [40, 42]. One additional study examined overall rates of reliable change between measures of symptom change, functioning, and progress towards self-defined goals, but without examining the extent to which reliable change ratings for different outcome domains converged for individual service users.

### The Present Study

Building on previous research, this study examined the convergence of meaningful improvement ratings [46] between (a) two measures of internalizing symptoms; (b) two measures of psychosocial functioning; and (c) between aggregate ratings in the domains of symptoms, functioning, and progress towards self-defined goals in a sample of adolescents aged 12–18 years with moderate or severe depression and/or anxiety symptoms who accessed routine specialist mental health care. Meaningful improvement was defined as reliable improvement on a standardized scale, and as meaningful improvement on an idiographic, goal-based outcome measure. The study used naturalistic data obtained through the routine administration of self-report measures that are widely used across a range of mental health settings in England [47].

## Methods

### General Setting

This study was a post-hoc analysis of naturalistic outcome data collected by specialist child and adolescent mental health services in England between 2011 and 2015, and collated by the Child Outcomes Research Consortium (CORC)^1^ as part of NHS England’s Children and Young People’s Improving Access to Psychological Therapies (CYP IAPT) service transformation initiative [34, 48]. Services could select from several standardized youth- and/or parent-reported measures of symptoms and functioning, as well as a personalized measure of progress towards self-defined goals [49].

### Participants and Process

The full CYP IAPT dataset included 96,325 case records of which 23,373 cases attended at least one appointment following the initial assessment [34]. For inclusion in this analysis, cases had to be aged 12–18 years and have moderate to severe anxiety or depression symptoms as indicated by clinicians on the Current View tool [50]. This was true for 15,352 case records. As this study examined rates of meaningful improvement, cases rated as showing only mild anxiety or depression symptoms at first assessment were not included, to ensure sufficient scope for improvement.

**Fig. 1:**
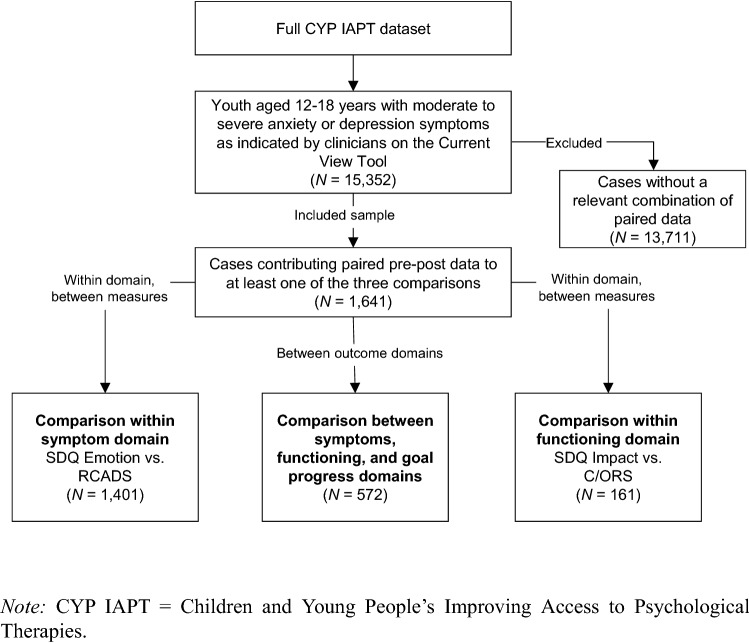
Flowchart of the analytical process and sampling

Cases also needed to have *paired data* on a relevant combination of outcome measures, that is, they needed to have completed the target measures on at least two occasions. Cases were required to either have paired data on both internalizing symptom measures (*N* = 1401), or on both functioning measures (*N* = 161); or on at least one symptom measure, one functioning measure, and the goal-based outcome measure (*N* = 572) to be included. This formed an overall analytical sample of 1641 cases. Because services were not mandated to use specific measures, different tools were administered in a variety of combinations, leading to a high amount of missing data on some measures. The breakdown of the study sample into the three analytical subsamples is illustrated in Fig. 1.

Of the 1641 cases included, 75.1% were female, 87.4% identified as white British, and the mean age was 14.8 years (SD = 1.47). On the Current View, 49.5 and 22.1% of the included sample were rated as showing moderate or severe anxiety symptoms, respectively; and 55.6 and 11.6%, respectively, were rated as showing moderate or severe depression symptoms (see Table 1). Data originated from 60 specialist child and adolescent mental health services and the average contact length was 31.0 weeks. Compared with cases that were excluded due to missing data, included cases were more likely to be female, *χ*^2^(1) = 79.05, *p* < 0.001; and to identify as white, *χ*^2^(1) = 23.62, *p* < 0.001. Youth in the included sample were slightly more likely to be classified as severely anxious *χ*^2^(1) = 28.27, *p* < 0.001, or severely depressed, *χ*^2^(1) = 21.31, *p* < 0.001. There was no difference in mean age between the included and excluded samples, t(15,350) = − 0.83, *p* = 0.41.

**Table 1:** Characteristics of the included and excluded samples

Characteristics	Excluded sample	Included sample			
		Total included sample	Symptom comparison	Functioning comparison	Domain comparison
	*N* = 13,711 (%)	*N* = 1641 (%)	*N* = 1401 (%)	*N* = 161 (%)	*N* = 572 (%)
Sex (% female)	69.3	75.1	74.9	73.3	75.7
Ethnicity (% White British)	84.4	87.4	87.5	85.0	86.2
Current view anxiety rating					
% moderate anxiety	49.7	49.5	49.3	41.0	51.7
% severe anxiety	20.5	22.1	21.8	26.3	22.4
Current view depression rating					
% moderately depressed	54.93	55.6	55.2	63.8	55.4
% severely depressed	10.0	11.6	12.32	11.3	9.1
Current view ratings for co-occurring problems (moderate or severe)					
% self-harm	24.6	26.1	26.8	25.0	25.7
% PTSD	17.1	13.0	13.3	9.5	11.0
% OCD	12.1	12.4	12.1	8.8	13.6
% CD or ODD	11.3	6.0	6.1	6.8	4.6
% eating disorder	10.1	9.0	9.1	8.9	5.8
% ADHD/hyperactivity	8.6	5.7	5.5	6.0	6.0
% psychosis or bipolar disorder	8.3	5.6	5.5	6.2	4.8
% substance use	4.0	1.6	1.7	4.1	1.3

	*M* (SD)	*M* (SD)	*M* (SD)	*M* (SD)	*M* (SD)
Age (in years)	14.7 (1.54)	14.8 (1.47)	14.8 (1.47)	14.9 (1.36)	14.8 (1.46)
Mean contact length (weeks)^a^	N/A	31.0 (20.39)	31.0% (20.63)	36.7 (21.46)	31.2 (20.02)
No. of services	77	60	60	32	53

*ADHD* attention deficit hyperactivity disorder, *CD* conduct disorder, *OCD* obsessive–compulsive disorder, *ODD* oppositional defiant disorder, *PTSD* post-traumatic stress disorder

^a^Length of contact was computed based on the dates of the very first and very last assessment completed on the RCADS, the SDQ or the C/ORS

For 24.6% of the included sample, clinician ratings on the Current View indicated at least moderate self-harm alongside symptoms of anxiety or depression. In addition, 33.5% of youth were rated as having moderate to severe co-occurring difficulties in at least one other problem area, with symptoms of post-traumatic stress disorder (13.0%) and obsessive–compulsive disorder (12.4%) being the most common co-occurring presenting problems (see Table 1). The naturalistic dataset does not provide information on the primary diagnosis. As such, it was not possible to establish whether youth with co-occurring problems were treated primarily for anxiety or depression. Similarly, the Current View tool does not capture whether moderate or severe symptoms in any given problem area were accompanied by a formal clinical diagnosis.

Young people received specialist routine care as provided by the 60 child and adolescent mental health services that contributed data to this study. Treatment approaches were not consistent across the sample, but varied according to the modalities and protocols used by each service, and the needs of individual cases. Data on the type of treatment received was available for 50.2% of the included sample. The most common type of therapy received by youth was cognitive behavioural therapy (65.3%). Other treatments included systemic family therapy (16.6%), psychodynamic or psychoanalytic psychotherapy (10.6%), brief solution-focused therapy (9.2%) and interpersonal psychotherapy (8.1%).

### Ethical Review

As this study was a secondary analysis of routinely collected data, ethical review was not required [51].

### Measures

#### Current View [50]

The current view is a screening tool to be completed by clinicians at first contact to provide a snapshot view of a case profile. The tool captures presenting problems, complexity factors and contextual problems, but not formal diagnoses. Clinicians are instructed to complete the Current View by drawing on all relevant information they have available at the time of tool completion. This includes information obtained through conversations during intake and initial assessment, information shared in the referral process (e.g., by other health professionals, teachers, or social workers), and scores from self- or clinician-reported outcome measures [50]. Presenting problems are assessed via 30 problem descriptions that map onto ICD-11 diagnostic criteria relevant to children and adolescents [52]. Based on the information available to them, clinicians rate the perceived severity of distress and impairment for each problem on a scale from 0 (none) to 3 (severe). Anxiety is identified via six problem descriptions that respectively indicate symptoms of separation anxiety, social phobia, generalized anxiety, panic disorder, agoraphobia, and specific phobia. Depression is identified through problem description number 9: “Depression/low mood (Depression).” As the naturalistic dataset did not contain information about the formal diagnoses assigned by the treating clinicians, the Current View problem descriptions were used as a proxy in this study to appraise the baseline severity of anxiety, depression, and co-occurring difficulties.

#### Revised Children’s Anxiety and Depression Scale (RCADS)—Total Score [53]

The RCADS is a 47-item self-report scale for 8–18-year-olds, measuring the frequency of symptoms associated with depression and anxiety. Young people are asked to state how frequently they experience each symptom, using a four-point Likert scale ranging from 0 (never) to 3 (always). A recall period is not specified. The RCADS consists of six subscales measuring symptoms of major depressive disorder (ten items), generalized anxiety disorder (six items), separation anxiety disorder (seven items), social phobia (nine items), panic disorder (nine items), and obsessive–compulsive disorder (six items), in line with DSM-IV dimensions [54]. Subscale scores can be summed to compute overall anxiety, depression, and internalizing symptom scores. The RCADS has demonstrated good internal consistency, test–retest reliability, and construct validity [53, 55–58].

#### Strengths and Difficulties Questionnaire (SDQ)—Emotional Problems Subscale [59, 60]

The SDQ is a 25-item self-report measure of psychosocial difficulties in children and adolescents aged 4–16 years. Respondents are asked to rate problem descriptions on a 3-point scale, from 0 (“not true”) to 2 (“certainly true”). An assessment version of the SDQ to be used at first measurement asks about psychosocial problems with reference to the past six months. A follow-up version of the SDQ, to be used at subsequent measurement time points, enquires about psychosocial difficulties in the past month. The SDQ includes a five-item emotional problems subscale that captures unhappiness, worries, clinginess*,* fears, and somatic symptoms. The five items can be summed to obtain a total subscale score ranging from 0 to 10. While the SDQ as a whole has been widely used and validated with regards to its internal consistency, test–retest reliability, construct validity and predictive validity [61–64], internal consistency for the emotional symptoms subscale has been shown to be questionable, with a Cronbach’s Alpha of 0.66 [62].

#### Strengths and Difficulties Questionnaire (SDQ)—Impact Supplement [65]

The SDQ Impact Supplement assesses the impact of psychosocial difficulties captured by the regular SDQ on a young person’s daily life. It probes into the duration and degree of distress, and into the impact on home life, friendships, classroom learning and leisure activities. The assessment version of the SDQ Impact enquires about the impact of psychosocial difficulties overall, while the follow-up version enquires about the impact of psychosocial difficulties during the past month. The five items on distress and impact are scored on a 3-point scale from 0 (“not at all/only a little”), to 2 (“a great deal”) and summed to compute a total score ranging from 0 to 10. The measure’s developers report good internal consistency, with a Cronbach’s Alpha of 0.81 [62].

#### Child Outcome Rating Scale (CORS) and Outcome Rating Scale (ORS)—Total Score [66]

The CORS and the ORS are four-item self-report measures of general distress and psychosocial functioning. The CORS was designed for use with children aged 6–12 years and is more child-friendly in wording and layout than the ORS, which was designed for youth aged 13 and older [67]. Both versions consist of four items that cover personal well-being, interpersonal functioning (e.g., with regards to family and close relationships), social functioning (e.g., at school or work), and overall well-being. The CORS asks about how things are going in general, while the ORS enquires about how things have been over the past week. Responses to each question are recorded as markings on a 10 cm visual analogue scale. Scoring is done by measuring the length between the starting point of the visual analogue scale and the marker, and by converting the distance from centimetres into score points (i.e., ten is the highest-possible score). A total score is computed by summing the four subscale scores. Validation studies have reported good internal consistency (between *α* = 0.81 and 0.97) [68–70], but mixed findings for test–retest reliability (*r* = 0.66–0.81) [66, 68]. For this analysis, CORS and ORS scores were combined into a composite score.

#### Goals Based Outcomes (GBO) [71]

The GBO tool is a personalized outcome measure, designed primarily with clinical utility in mind [72]. Young people can define a number of treatment goals, the top three of which are typically used for outcomes reporting [73]. Progress is rated periodically on a scale from 0 (“goal not at all met”) to 10 (“goal reached”), with young people indicating how they would rate their progress on the given day [74]. Only goals defined primarily by adolescents themselves were considered for this analysis. Data on the reliability of the GBO is not currently available [75].

### Statistical Analysis

#### Assessing Meaningful Change

The criterion used in this study to determine the salience of individual-level change was the reliable change index (RCI) [76] for standardized measures, and a meaningful change index for the GBO. The RCI determines the amount of change required to demarcate improvement beyond fluctuations attributable to measurement error [76, 77]. The RCI is calculated by dividing the difference between T1 and T2 scores by the standard error of the difference between the two measurements (see Online Appendix for details). We computed the RCI based on the standard deviation of the mean T1 score and the measure’s internal consistency at T1 in the subsample that contributed paired data on the relevant measure to any of the three comparisons. The SDQ Emotion and SDQ Impact demonstrated questionable internal consistency (α = 0.64 and 0.65, respectively). Internal consistency was good on the C/ORS (α = 0.87) and excellent on the RCADS (α = 0.95). To be considered as reliably improved on a given measure, individuals had to demonstrate a pre-post score difference exceeding RCI thresholds of 15.68 for the RCADS, 3.62 for the SDQ Emotions, 3.97 on the SDQ Impact, and 8.40 on the C/ORS (Table 2).

**Table 2:** Parameters used to determine the RCI for each standardized measure in the study sample

Measure	N	*M*_T1_ (SD)	*M*_T1_–*M*_T2_ (SD)	Cronbach alpha	RCI/MCI threshold
SDQ emotion	1577	7.13 (2.16)	− 1.53 (2.62)	0.64	3.62
RCADS	1427	69.85 (25.30)	− 18.45 (26.91)	0.95	15.68
SDQ impact	636	4.32 (2.43)	− 1.71 (2.79)	0.65	3.97
C/ORS	198	20.50 (8.45)	6.59 (9.55)	0.87	8.40

The reliable change index for the GBO has previously been defined as a movement by at least 2.45 along the goal progress scale, where progress scores are aggregated across the three goals [43]. However, as service users were free to define fewer than three goals in CYP IAPT, the incidence of missing data was high. We chose to compute an alternative meaningful change index that would use all available GBO data, without requiring complete measurements on all three goals. Meaningful change on the GBO was defined as youth showing an improvement by at least three scale points on any completed goal, without equivalent deterioration on any other available goal [33]. For the sake of brevity, we will use the shorthand term “improved” to describe both reliable improvement and meaningful improvement.

We conducted a series of bivariate comparisons of improvement between the SDQ Emotion and the RCADS within the symptom domain (*N* = 1401); between the SDQ Impact and the C/ORS within the functioning domain *(N* = 161)*;* and between pairs of outcome domains (*N* = 572). We also conducted a multivariate comparison of improvement rates between the three outcome domains of symptoms, functioning, and goal progress. Composite improvement indices were computed for the symptom and functioning domains by defining as reliably improved those who showed reliable improvement on at least one of the two measures within each domain, and no reliable deterioration on the other (see Table A2 in the Online Appendix).

Since few cases showed reliable or meaningful deterioration (ranging from 1.9 to 7.0%, see Table A1 in the Online Appendix), cross tables of dichotomized improvement ratings were computed, distinguishing only between improvement versus no improvement (including deterioration) to avoid cell sample sizes below the reportable minimum [78]. We computed McNemar’s test of correlated proportions [79] to assess the likelihood of no agreement, and Cohen’s Kappa for chance-corrected agreement to estimate the level of agreement in bivariate comparisons [80]. Fleiss’ kappa was computed to assess agreement across all three domains [81].

#### Assessing Discrepancies in Assessment Timelines for the Standardized Measures

As this study used naturalistic data, we expected outcome assessment timelines to vary between individuals, as well as within individual cases with regard to different measurement instruments. We conducted descriptive analysis to examine the mean and median time between the first assessment (hereafter “T1”) and last assessment (hereafter “T2”) for each standardized measure. A Wilcoxon signed rank test was conducted to assess the significance of any observed differences in median time for the pairs of symptom and functioning measures, respectively. Univariate logistic regression was conducted to examine whether chances of reliable improvement increased with increasing time elapsed between T1 and T2. Data on assessment timelines for the GBO were not available.

## Results

### Comparing Improvement Within the Symptom and Functioning Domains

Figures illustrating the results below are included in the Online Appendix (page 4).

#### Convergence of Improvement Ratings Between Symptom Measures

In the sample with paired data on both internalizing symptom measures (*N* = 1401), reliable improvement rates were considerably higher on the RCADS (49.3%) than on the SDQ Emotion (22.9%, see Table 3). At an individual level, improvement ratings were discrepant in close to one third of cases. Of all cases who did not improve on the SDQ Emotion, more than a third (37.4%) did improve on the RCADS. Of the cases that improved on the RCADS, 58.5% failed to improve on the SDQ Emotion. McNemar’s test of correlated proportions showed a significant difference in improvement ratings [χ^2^(1) = 312.6; *p* < 0.001], and Cohen’s kappa indicated fair agreement (κ = 0.37; *p* < 0.001) between the two measures. Only 20.5% of cases improved on both symptom measures.

**Table 3:** Disagreement between measures and domains (bivariate comparisons)

First measure/domain		Second measure/domain		
		Not improved	Improved	Total
		*n* (%)	*n* (%)	*n* (%)
*Within the symptom domain*				
RCADS				
SDQ emotion	Not improved	676 (48.3)	404 (28.8)	1,080 (77.1)
	Improved	34 (2.4)	287 (20.5)	321 (22.9)
	Total	710 (50.7)	691 (49.3)	1401 (100)
*Within the functioning domain*				
C/ORS				
SDQ impact	Not improved	72 (44.7)	44 (27.3)	116 (72.0)
	Improved	25 (15.5)	20 (12.4)	45 (28.0)
	Total	97 (60.2)	64 (39.8)	161 (100)
*Across the symptom, functioning, and goal progress domains (paired comparisons)*				
Functioning				
Internalizing symptoms	Not improved	270 (47.2)	56 (9.8)	326 (57.0)
	Improved	137 (24.0)	109 (19.1)	246 (43.0)
	Total	407 (71.2)	165 (28.8)	572 (100)
Goal progress				
Internalizing symptoms	Not improved	127 (22.2)	199 (34.8)	326 (57.0)
	Improved	45 (7.9)	201 (35.1)	246 (43.0)
	Total	172 (30.1)	400 (69.9)	572 (100)
Goal progress				
Functioning	Not improved	139 (24.3)	268 (46.9)	407 (71.2)
	Improved	33 (5.8)	132 (23.1)	165 (28.8)
	Total	172 (30.1)	400 (69.9)	572 (100)

#### Convergence of Improvement Ratings Between Functioning Measures

**Fig. 2:**
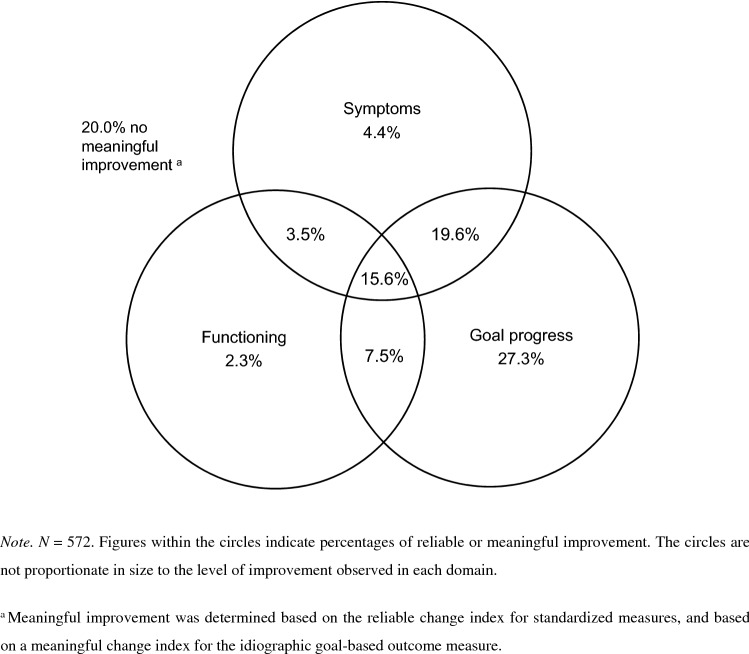
Venn diagram of meaningful improvement.^a^ Across all three outcome domains

In the sample with paired data on both functioning measures (*N* = 161), reliable improvement was considerably higher on the C/ORS (39.8%) than on the SDQ Impact (28.0%, Table 3). At an individual level, improvement ratings were discrepant in 42.9% of cases. Of all cases improving on the C/ORS, over two thirds (68.8%) showed no improvement on the SDQ Impact. At the same time, of the cases that improved on the SDQ Impact, 55.6% did not improve on the C/ORS (see Fig. 2). McNemar’s test of correlated proportions [*χ*^2^(1) = 5.23, *p* = 0.030] indicated marginally significant disagreement. Cohen’s kappa (κ = 0.06, *p* = 0.224) was not statistically significant, likely due to the small sample size. Only 12.4% of cases improved on both functioning measures.

### Comparing Improvement Across Domains

In the sample with paired data on at least one symptom measure, one functioning measure, and the goal-based outcome measure (*N* = 527), 69.9% of adolescents meaningfully improved their goal progress, 43.0% improved their internalizing symptoms, and 28.9% improved their functioning (see Table A1 in the Online Appendix). Figures illustrating these results are included in the Online Appendix (pages 4–5).

#### Comparing Improvement Between the Symptom and Functioning Domains

One third (33.7%) of cases showed discrepant improvement ratings across the symptom and functioning domains. Of all cases that improved in the symptom domain, 55.7% did not improve their functioning (Table 3). In turn, of those improving their functioning, 33.9% did not improve their symptoms. McNemar’s test showed a significant difference in improvement ratings between the symptom and functioning domains [*χ*^2^(1) = 33.99; *p* < 0.001]. Cohen’s kappa indicated fair agreement (κ = 0.28; *p* < 0.001). Only 19.1% of cases improved in both domains.

#### Comparing Improvement Between the Symptom and Goal Progress Domains

Around 42.7% of cases showed discrepant improvement ratings across the symptom and goal domains. Of all those who improved their goal progress, 49.8% showed no improvement in internalizing symptoms, while of those not improving on the GBO, 26.2% still improved their symptoms. McNemar’s test of correlated proportions showed a significant difference in improvement ratings between the symptom and goal progress domains [*χ*^2^(1) = 97.20; *p* < 0.001] while Cohen’s kappa indicated slight agreement (κ = 0.19; *p* < 0.001). A third of cases (35.1%) showed improvement in both domains.

#### Comparing Improvement Between the Functioning and Goal Progress Domains

More than half of cases (52.6%) showed discrepant improvement ratings across the functioning and goal domains. Of all cases improving their goal progress, 67.0% failed to improve their functioning. In turn, of those who did improve their functioning, 20.0% showed no improvement in goal progress. McNemar’s test of correlated proportions showed a significant difference in improvement ratings between the functioning and goal progress domains [*χ*^2^(1) = 183.47; *p* < 0.001]. Cohen’s kappa (κ = 0.10; *p* < 0.001) indicated slight agreement. Only 23.1% of cases improved in both domains.

#### Comparing Improvement Between the Symptom, Functioning and Goal Progress Domains

When comparing rates of meaningful improvement across all three domains, 64.5% of cases showed discrepant ratings. Of all 572 cases considered, 20.0% did not show improvement in any domain, 27.3% improved exclusively in the goal progress domain, 19.6% improved their symptoms and goal progress but not their functioning, and 15.6% improved across all three domains [with 2–8% of cases showing other combinations of change (Table 4, Fig. 2)]. Fleiss’ kappa showed only slight agreement in improvement ratings across the three domains (κ_f_ = 0.14; *p* < 0.001).

**Table 4:** Disagreement between symptoms, functioning, and goal progress

	Functioning	Goal progress	
		Not improved	Improved
Internalizing symptoms			
Not improved	Not improved	114 (19.9%)	156 (27.3)
	Improved	13 (2.3%)	43 (7.5%)
Improved	Not improved	25 (4.4%)	112 (19.6%)
	Improved	20 (3.5%)	89 (15.6)

*N* = 572 (100%)

### Assessment Timelines for the Standardized Measures

Within the symptom measure comparison sample (*N* = 1401), the mean amount of time elapsed between T1 and T2 was 29.3 weeks for the RCADS (SD = 20.1; median = 25 weeks), and 28.6 weeks for the SDQ Emotions (SD = 19.4; median = 24.9 weeks). A Wilcoxon signed rank test showed no significant difference between the two measures (Z = 0.433, *p* = 0.67). For 78.4% of the sample, the number of weeks elapsed was identical on both measures (see Online Appendix). Within the functioning measure comparison sample (*N* = 161), the mean amount of time elapsed between T1 and T2 was 28.8 weeks (SD = 20.1; median = 24.9 weeks) for the SDQ Impact, and 20.65 weeks (SD = 17.3; median = 16.0 weeks) for the C/ORS. A Wilcoxon signed rank test showed a significant difference between the two measures (Z = − 5.261, *p* < 0.001). The amount of time elapsed was identical across the two measures for only 14.9% of the sample. Given the longer average assessment period for the SDQ Impact, it might be expected that this measure captured higher rates of reliable improvement. However, a univariate logistic regression showed no significant association between increasing length of time between assessments and the odds of achieving reliable improvement on the RCADS, the SDQ Emotions or the C/ORS (see Online Appendix), and only a marginally significant association for the SDQ Impact (OR 1.02; *p* = 0.046; 95% CI 1.00–1.03).

## Discussion

This study assessed levels of meaningful improvement among adolescents with moderate or severe anxiety and/or depression symptoms across five commonly used self-report measures of internalizing symptoms, functioning, and progress towards self-defined goals; and examined the convergence of improvement ratings within and between these three outcome domains. We found considerable disagreement between measures and domains. The two symptom measures yielded discordant ratings for close to one third of cases, and the two functioning measures for over 40%. Similar levels of bivariate discordance were observed between the three domains of symptoms, functioning, and goal progress. There were discrepancies in 64.5% of cases when considering all three domains simultaneously. Improvement rates were highest in the goal progress domain (69.9%), and lowest in functioning (28.9%). Improvement was observed consistently across all three domains for only 15.6% of cases.

Within the symptom domain, the RCADS showed higher levels of reliable improvement (49.3%) than the SDQ Emotion (22.9%), although there was no significant difference in average assessment timelines between the two measures. Both measures track internalizing symptoms, but the RCADS provides a more detailed assessment of symptoms related to major depression (via 10 items), and to five anxiety disorders (via 6–9 items for each disorder). The SDQ Emotion consists of just one item measuring low mood; three items capturing fears, worries, and clinginess; and one item capturing somatic symptoms. Our finding is in line with previous research suggesting that more broadly defined measures are less likely to capture treatment effects than more specific measures [82], which may need to be taken into account when choosing measures for clinical or research use [47, 72, 83].

Within the domain of functioning, the C/ORS showed higher levels of reliable improvement than the SDQ Impact (39.8 and 28.0%, respectively). Both measures are of comparable length and cover psychosocial functioning in the family, peer, and school context. Both also enquire about global notions of distress or well-being. But while the SDQ Impact probes into functional impairment caused by mental health problems, the C/ORS asks young people how well they were generally doing. Although functioning measures probing about disorder-specific impairment have been described as more sensitive to change than generic measures [84], the problem-specific SDQ Impact displayed lower levels of change than the generic C/ORS. This was in spite of the average period between the T1 and T2 assessments being longer for the SDQ Impact than for the C/ORS, which would have allowed more time for functional improvements to become apparent. This discrepancy may be due to differences in response scales. The SDQ Impact uses a four-point Likert scale, while the C/ORS uses a ten-point continuous scale that may be better able to capture nuanced change. The observed discrepancy may also be driven by the differential internal consistencies of both measures, which led to a more conservative reliable change threshold for the SDQ Impact.

Our finding of limited convergence between reliable improvement in symptoms and functioning, and of less observed change in functioning is consistent with previous research [39, 40]. Associations between measures of depression symptoms and functioning may vary based on the types of symptoms assessed, as some symptoms may explain a larger variation in functional impairment than others [85]. In addition, several studies in adult populations have found change in social and global functioning to lag behind change in depressive symptoms [41]. Symptom change may be an early sign of treatment response, while functioning may be slower to manifest but could indicate deeper or more wide-reaching impact. Another possible reason for the lower rates of reliable improvement observed in functioning may be that functioning measures for young people have received less attention from psychometricians, compared with symptom measures. They tend to have weaker psychometric properties, and possibly weaker sensitivity to change [39, 41, 86]. Another possible explanation is that 83% of cases considered for the cross-domain comparison had paired data on the SDQ Impact but not on the C/ORS. The comparatively low rate of reliable improvement in functioning may therefore be driven by the comparatively low rates of improvement on this specific measure, which further highlights the importance of careful measure selection.

Meaningful improvement in goal progress was considerably higher than reliable improvement in internalizing symptoms and functioning. This was consistent with two previous studies comparing change between standardized and personalized measures for children and young people [42, 43]. It is also consistent with a number of studies evidencing the superior sensitivity to change of personalized measures in adult mental health [87–89]. While parent-reported GBO scores have been shown to correlate moderately with parent-reported SDQ total difficulty scores (*r* = 0.3–0.4) and clinician-rated functioning (*r* = 0.4–0.5), no significant correlation has yet been found for child-reported measures of goal progress and symptoms [43, 90]. The GBO may capture changes that are uniquely different from those assessed by standardized measures of symptoms and functioning. For reasons related to the structure of the naturalistic dataset, it was not possible to consider the qualitative content of goals defined by adolescents in this study. However, a previous study examining the content of young people’s self-defined goals found that they covered themes such as independence, confidence, self-reflection, communicating feelings, and understanding anger, which are not covered by commonly used standardized measures of symptoms or functioning [91]. Similarly, a recent study of drop-out in a treatment trial for adolescent depression reported that some young people ended treatment within three sessions, because they felt they had achieved their personal treatment goals, although this was not reflected by standardized outcome measures [92].

### Administrative and Clinical Implications

The outcome domains services choose to monitor, and the tools they select for this purpose have a bearing on who is judged to have realized a “good” outcome, and what service models warrant funding. Measurement tools purported to measure a similar concept cannot be assumed to be comparable even when a standardized indicator such as the RCI is computed to facilitate comparisons. Due to differential psychometric properties (e.g., reliability and sensitivity to change) and item content, apples may be compared with oranges. This can have critical real-world implications where these discrepancies are interpreted as true differences in clinician or service performance.

Our findings corroborate existing evidence that symptom change by itself does not represent a sufficient proximal indicator for overall treatment effectiveness, as it may over- or under-estimate change in other outcome domains. Our findings call for more multidimensional approaches to routine outcome measurement, but multidimensional approaches raise challenges for interpreting and reconciling conflicting results that are comparable to the challenges posed by combining data from multiple reporters [93]. While the aggregation of reliable or meaningful improvement rates across measures and outcome domains provides a means of simplification and reduces the risk of missing change where it does occur, it fails to discriminate between cases that improve across all measures and/or domains, and cases that improve in only one. Such approaches may mask nuances that could help distinguish usual from best practice.

Given the lack of comparability between different outcome measures and outcome domains, mental health systems and services should seek to (a) track several meaningful outcome domains to gain a more holistic picture of the changes achieved, and (b) consider applying standards for outcome measurement, which are beginning to emerge. Our findings demonstrate that a common, harmonized approach to outcome measurement is needed if outcomes are to be compared fairly across mental health services and settings. A global standard set of outcomes for child and youth anxiety and depression has recently been developed under the lead of the International Consortium for Health Outcomes Measurement (ICHOM) [21]. The standard set recommends tracking change in the domains of symptoms, functioning, and suicidal thoughts and behaviour, as a minimum, when providing routine care for youth with anxiety or depression. The standard set further recommends a suite of seven feasible, valid, and reliable measurement instruments (including a short version of the RCADS), and suggests a timeline for outcome measurement.

The high rate of meaningful improvement on the GBO indicates that many young people experience change that is not reflected by standardized symptom and functioning measures. Based on this finding, services should consider administering personalized measures alongside standardized ones, so as not to miss idiographic treatment impact. Goal setting has demonstrated clinical value, in addition to being a flexible means of progress tracking, in terms of improving retention in treatment [94], and strengthening adolescents’ perceived self-awareness and problem-solving ability [95].

### Future Research

Further research is needed that examines the sensitivity to change of commonly used outcome measures, and that compares the magnitudes of change that each instrument can reliably detect at an individual level. There are no established cut-off criteria for establishing when a scale is sufficiently sensitive to change [21, 96]. The International Society for Quality of Life Research (ISOQOL) recommends that a measure “should have evidence of responsiveness, including empirical evidence of changes in scores consistent with pre-defined hypotheses” (p. 1901) [97]. One promising avenue for future research is to explore the *minimally important difference*, that is the minimum magnitude of change that is perceived as meaningful by service users and their families on a given scale [96, 98]. Reporting rates of minimally important difference that are anchored in stakeholder perceptions alongside rates of reliable improvement that are statistically derived could help with interpreting and contextualizing change metrics.

It is currently not well understood why treatment effectiveness is more difficult to evidence in functioning, compared with symptom change [39, 86, 99]. More research is needed to assess the validity, reliability, and sensitivity to change of youth-reported functioning measures, to understand whether brief scales such as the SDQ Impact and C/ORS provide the best possible avenue for tracking treatment response in clinical practice, or whether more granular measures are needed. There would be value in comparing change trajectories for symptoms and functioning over time, to explore whether outcomes in both domains are likely to converge at certain time points or for specific subgroups.

Personalized measures may show a higher sensitivity to change, because they are tailored to capturing change in the problems most salient to service users, and that treatment should ideally focus on [71, 73, 100]. However, the high levels of change measured by the GBO might also stem from services defining goals that are “too easy” to achieve [100]. More research involving children, adolescents, families, and clinicians, is needed to confirm that the currently used threshold for meaningful change is appropriate [75]. In addition, future research should examine the GBO’s sensitivity to change and convergence with other measures in relation to specific goal themes, and to ascertain the psychometric properties of goals and other personalized measures.

### Limitations

The above-mentioned findings should be considered in the context of several limitations. First, the dataset analysed for this study has been described elsewhere as an example of naturalistic data that are *flawed*, *uncertain*, *proximate,* and *sparse* (“FUPS”) [83]. There was a high incidence of missing data, which could not be explained by the observed variables, hence limiting possibilities for data imputation. Second, the two assessment time points included in this dataset provide a snapshot view of the change achieved. Due to the high number of cases lost to follow up, change trajectories could not be examined in detail across several time points. In addition, there was considerable variation in assessment time points for different measures, especially for the two functioning measures, which may have added to the lack of convergence observed. Third, as outlined above, data on the qualitative content of the goals captured through the GBO were not available. As such, it was not possible to examine the extent to which self-defined goals covered similar content as the standardized measures examined in this study. Similarly, no information was available about the extent to which these goals were clinically relevant and achievable within the time course of specialist mental health support. As such, it was not possible to assess how goal characteristics may have influenced the high rates of meaningful improvement reported on the GBO in this study.

For the comparison across domains, composite reliable improvement indices were computed that pulled available data from the four symptom and functioning measures. As suggested above, the differential reliability of these measures meant that different magnitudes of change were required by each tool to exceed the reliable change threshold. Reliable change ratings that informed the composite metric for functioning were pulled exclusively from the SDQ Impact for 83% of the cases considered (see Table A2, in the Online Appendix). Given that the SDQ Impact indicated only about half the amount of reliable change as the C/ORS in the within-domain comparison, the cross-domain comparisons might have shown less discrepant results (due to higher rates of improvement in the functioning domain), had a larger share of functioning ratings been informed by the C/ORS.

Another limitation of this study is that we could not consider levels of convergence in relation to deterioration ratings, as the number of cases showing reliable or meaningful deterioration on the relevant measures were too small. Distinguishing not just between improvers and non-improvers, but also between those not showing any change and those showing deterioration might have led to even higher levels of discrepancy between measures and outcome domains, as differential sensitivity to change might have led to diverging rates of deterioration as well as improvement.

## Conclusions

Routinely collected outcomes data is a crucial tool for strengthening service effectiveness. In countries where routine outcome monitoring is well established it informs decision-making about service organization, allocation of funds, and policy priorities in child mental health [20]. For service evaluations and benchmarking to be fair and meaningful, metrics and measurement approaches must be comparable. This study suggests that reliable or meaningful change indicators are not inherently comparable if drawn from different outcome domains or measures. Aggregating change into a single composite indicator risks foregoing the benefits of multidimensional outcome measurement by masking differences in treatment impact on different domains. Making maximum use of all available data and exploring what might drive inconsistencies between these data would enable more nuanced insights into what treatments work; for whom; and with regards to which outcome [93]. Emerging global standards for routine outcome measurement aim to promote harmonization, but require widespread uptake by mental health systems and services to be successful. Such initiatives can help focus resources and interest on a set of recommended measures, which can then be calibrated thoroughly against one another, and for which reliability, validity, and sensitivity to change can be studied in detail [101]. With practice-based evidence increasingly driving decisions about care, the stakes are high and ambiguity in outcomes reporting must be avoided.

## Summary

Anxiety and depression are prevalent mental health problems in adolescence. Routinely collected outcome data is key to providing an effective and evidence-based clinical response. Those wishing to compare outcomes achieved in different services or systems are typically faced with data obtained through inconsistent measurement approaches. Mitigation strategies include the comparison of symptom-focused metrics, such as rates of remission or recovery, or the aggregation of results obtained from different measures or outcome domains into a comparable core metric, such as the reliable change index. The implications of either approach for judging treatment success are not well understood.

This study compared meaningful change between two self-report measures of internalizing symptoms; two brief self-report measures of psychosocial functioning; and between the outcome domains of symptoms, functioning and progress towards self-defined goals. Meaningful change was defined based on the reliable change index for standardized scales, and based on a meaningful change index for the idiographic goal-based outcome measure. The data originated from a naturalistic sample of 1,641 adolescents with moderate or severe anxiety and/or depression symptoms who were treated across 60 child and adolescent mental health services in England. Improvement was observed consistently across all three outcome domains in only 15.6% of cases. A comparison of reliable improvement ratings between domains revealed that 24% of young people showed reliable improved in symptoms, but no improvement in functioning. Inversely, 34.8% of young people showed meaningful progress towards self-defined goals, but no improvement in symptoms.

Focusing outcome reporting exclusively on symptom change risks over- or under-estimating actual treatment effectiveness, while aggregating data from several outcome domains into a single metric can mask informative differences in the number and type of outcomes showing improvement. Instead, mental health systems and services should consider assessing multiple outcomes to gain a more balanced picture of the changes achieved, and should draw on emerging standards for the selection of outcome domains, measurement instruments, and assessment time points, to ensure that their data can add to a growing, harmonized evidence base that allows for meaningful comparisons.

## Supplementary Information

Below is the link to the electronic supplementary material.

Supplementary file 1 (DOCX 177 KB)

## Footnotes

1 CORC is a professional learning collaboration.
