# Supplementary material for: Are We Comparing Apples with Oranges? Assessing Improvement Across Symptoms, Functioning, and Goal Progress for Adolescent Anxiety and Depression
Source: Child Psychiatry Hum Dev. 2021 Apr 7;53(4):737–53. doi: 10.1007/s10578-021-01149-y (PMC9287244; doi:10.1007/s10578-021-01149-y)
Supplement: Supplementary file 1 — (DOCX 177 KB) [file 10578_2021_1149_MOESM1_ESM.docx]

Child Psychiatry & Human Development

Are We Comparing Apples with Oranges? Assessing Reliable Improvement Across Symptoms, Functioning, and Goal Progress for Adolescent Anxiety and Depression

**ONLINE RESOURCE 1**

Karolin Rose Krause, Rosie Singleton, Julian Edbrooke-Childs, Miranda Wolpert

**Corresponding Author**: Karolin Rose Krause, Research Department of Clinical, Educational and Health Psychology, University College London, Gower Street, London WC1E 6BT, United Kingdom; karolin.krause.16@ucl.ac.uk.

**Table of Contents**

The Reliable Change Index 3

Interpretation of Cohen’s Kappa 3

Supplementary Tables and Figures 4

References 7

**Table of Tables**

Supplementary Table 1. Levels of Reliable Change 4

Supplementary Table 2. Composition of the Aggregate Reliable Change Metric for Symptoms and Functioning Domains 4

Supplementary Table 3. Length of the Assessment Period by Standardized Outcome Measure 7

Supplementary Table 4. Percentage of Cases with the Specified Time Lapse for the Given Measurement Instrument 7

**Table of Figures**

Supplementary Figure 1. Disagreement Between the Symptom Measures 5

Supplementary Figure 2. Disagreement Between the Functioning Measures 5

Supplementary Figure 3. Disagreement Between the Symptom and Functioning Domains 5

Supplementary Figure 4. Disagreement Between the Symptom and Goal Progress Domains 6

Supplementary Figure 5. Disagreement Between the Functioning and Goal Progress Domains 6

The Reliable Change Index

The reliable change index (RCI) is calculated by dividing the difference between scores at first (T1) and second (T2) measurement by the standard error of the difference between the two measurements:

RC = $\frac{x_{1}-x_{2}}{S_{\text{diff}}}$

where x_1_ and x_2_ are an individual’s scores at T1 and T2, and S_diff_ is the standard error of the difference between these scores [1]. S_diff_ can be calculated from the standard error of measurement (S_E_) according to the following formula:

S_diff =_ $\sqrt{2}(S_{E})^{2}$

$S_{E}= S_{1}\sqrt{1}-r_{1}$

where S_1_ is the standard deviation of the score measured at T1 and, $r_{1}$ is the reliability of the measure, defined here as Cronbach’s Alpha, at T1. A magnitude of change exceeding 1.96 times the *S_diff_* is unlikely to be due to measurement error alone in more than 5% of cases. For each standardized measure, the RCI was computed based on the standard deviation of the mean T1 score and the measure’s internal consistency at T1, in the sub-sample that contributed paired data on the relevant measure to any of the three comparisons.

Interpretation of Cohen’s Kappa

Kappa values were interpreted as follows: κ ≤ 0 poor agreement, κ = 0.01–0.20 slight agreement, κ = 0.21–0.40 fair agreement, κ = 0.41–0.60 moderate agreement, κ = 0.61–0.80 substantial agreement, and κ = 0.81–1.00 almost perfect agreement [2].

Supplementary Tables and Figures

Supplementary Table 1. Levels of Reliable Change

| **Measure/outcome domain** | |  | **Reliable change** | | |
| --- | --- | --- | --- | --- | --- |
|  |  |  | **Deteriorated**  ***n* (%)** | **Unchanged**  ***n* (%)** | **Improved**  ***n* (%)** |
| Sample used for the comparison between measures within the symptom domain (*N* = 1,401) | |  |  |  |  |
|  | SDQ Emotion |  | 26 (1.9) | 1,054 (75.2) | 321 (22.9) |
|  | RCADS |  | 98 (7.0) | 612 (43.7) | 691 (49.3) |
|  |  |  |  |  |  |
| Sample used for the comparison between measures within the functioning domain (*N* = 161) | |  |  |  |  |
|  | SDQ Impact |  | 8 (5.0) | 108 (67.1) | 45 (28.0) |
|  | C/ORS |  | 7 (4.4) | 90 (55.9) | 64 (39.8) |
|  |  |  |  |  |  |
| Sample used for the comparison between the three domains of symptoms, functioning, and goal progress (*N =* 527) | |  |  |  |  |
|  | Symptoms |  | 31 (5.4) | 295 (51.6) | 246 (43.0) |
|  | Functioning |  | 19 (3.3) | 388 (67.8) | 165 (28.9) |
|  | Goal progress |  | 21 (3.7) | 151 (26.4) | 400 (69.9) |

*Note. N* = 1,641

Supplementary Table 2. Composition of the Aggregate Reliable Change Metric for the Symptoms and Functioning Domains

| **Domain** | ***N* (%)** |
| --- | --- |
| Symptoms | 572 (100.0) |
| Only SDQ Emotion data available and considered | 176 (31%) |
| Only RCADS data available and considered | 28 (5%) |
| Data from both measures available and considered | 371 (65%) |
|  |  |
| Functioning | 572 (100.0) |
| Only SDQ Impact data available and considered | 474 (83%) |
| Only C/ORS data available and considered | 34 (6%) |
| Data from both measures available and considered | 57 (10%) |

Supplementary Figure 1. Disagreement Between the Symptom Measures

|  |  |  |  |
| --- | --- | --- | --- |

*Note. N* = 1,401

Supplementary Figure 2. Disagreement Between the Functioning Measures

|  |  |  |  |
| --- | --- | --- | --- |

*Note. N =* 161

Supplementary Figure 3. Disagreement Between the Symptom and Functioning Domains

|  |  |  |  |
| --- | --- | --- | --- |

*Note. N* = 527

Supplementary Figure 4. Disagreement Between the Symptom and Goal Progress Domains

|  |  |  |  |
| --- | --- | --- | --- |

*Note. N* = 572.

Supplementary Figure 5. Disagreement Between the Functioning and Goal Progress Domains

|  |  |  |  |
| --- | --- | --- | --- |

*Note. N* = 572

Supplementary Table 3. Length of the Assessment Period (T1 to T2) by Measurement Instrument

| **Duration of assessment period between T1 and T2** | **RCADS**  **(*N* = 1,401)** | **SDQ Emotion**  **(*N* = 1,401)** | **SDQ Impact**  **(*N* = 161)** | **C/ORS**  **(*N* = 161)** |
| --- | --- | --- | --- | --- |
| Mean length in days (SD) | 205.3 (140.3) | 199.9 (135.6) | 201.5 (140.5) | 144.5 (121.0) |
| Min | 1 | 1 | 21 | 4 |
| Max | 818 | 818 | 672 | 546 |

Supplementary Table 4. Percentage of Cases with the Specified Time Lapse Between the T1 and T2 Assessments for the Given Measurement Instrument

| **Duration of assessment period between T1 and T2** | **RCADS**  **(*N* = 1,401)** | **SDQ Emotion**  **(*N* = 1,401)** | **SDQ Impact**  **(*N* = 161)** | **C/ORS**  **(*N* = 161)** |
| --- | --- | --- | --- | --- |
|  | **% (% cum)** | **% (% cum)** | **% (% cum)** | **% (% cum)** |
| Less than 6 weeks | 6.7 (6.7) | 7.1 (7.1) | 6.8 (6.8) | 21.74 (21.74) |
| 6-12 weeks | 11.5 (18.2) | 11.6 (18.8) | 15.5 (22.4) | 16.2 (37.9) |
| 12-36 weeks | 53.8 (72.0) | 54.5 (73.3) | 49.7 (72.1) | 47.2 (85.1) |
| 36-52 weeks | 15.1 (87.0) | 14.6 (87.9) | 14.3 (86.3) | 7.5 (92.6) |
| 52-76 weeks | 9,6 (96.7) | 9.2 (97.1) | 11.2 (97.5) | 7.5 (100.0) |
| 76-104 weeks | 3.0 (99.6) | 2.7 (99.8) | 2.5 (100.0) | — |
| More than 104 weeks | 0.4 (100.0) | 0.2 (100.0) | — | — |

References

1. Jacobson NS, Truax P (1991) Clinical Significance: A Statistical Approach to Defining Meaningful Change in Psychotherapy Research. J Consult Clin Psychol 59:12–19. https://doi.org/10.1037/0022-006X.59.1.12

2. Landis JR, Koch GG (1977) The Measurement of Observer Agreement for Categorical Data. Biometrics 33:159–174. https://doi.org/10.2307/2529310
